# Melatonin and 5-fluorouracil combination chemotherapy: opportunities and efficacy in cancer therapy

**DOI:** 10.1186/s12964-023-01047-x

**Published:** 2023-02-09

**Authors:** Alireza Mafi, Malihe Rezaee, Neda Hedayati, Sara Diana Hogan, Russel J. Reiter, Mohammad-Hossein Aarabi, Zatollah Asemi

**Affiliations:** 1 Department of Clinical Biochemistry, School of Pharmacy and Pharmaceutical Sciences, Isfahan University of Medical Sciences, Isfahan, Islamic Republic of Iran; 2 School of Medicine, Shahid Beheshti University of Medical Sciences, Tehran, Islamic Republic of Iran; 3 Tehran Heart Center, Cardiovascular Diseases Research Institute, Tehran University of Medical Sciences, Tehran, Islamic Republic of Iran; 4 School of Medicine, Iran University of Medical Science, Tehran, Islamic Republic of Iran; 5 Department of Public Health and Caring Sciences, Uppsala University, Uppsala, Sweden; 6 Department of Cell Systems and Anatomy, UT Health. Long School of Medicine, San Antonio, TX USA; 7 Research Center for Biochemistry and Nutrition in Metabolic Diseases, Institute for Basic Sciences, Kashan University of Medical Sciences, Kashan, Islamic Republic of Iran

**Keywords:** Melatonin, 5-fluorouracil, Combination therapy, Chemotherapy

## Abstract

Combined chemotherapy is a treatment method based on the simultaneous use of two or more therapeutic agents; it is frequently necessary to produce a more effective treatment for cancer patients. Such combined treatments often improve the outcomes over that of the monotherapy approach, as the drugs synergistically target critical cell signaling pathways or work independently at different oncostatic sites. A better prognosis has been reported in patients treated with combination therapy than in patients treated with single drug chemotherapy. In recent decades, 5-fluorouracil (5-FU) has become one of the most widely used chemotherapy agents in cancer treatment. This medication, which is soluble in water, is used as the first line of anti-neoplastic agent in the treatment of several cancer types including breast, head and neck, stomach and colon cancer. Within the last three decades, many studies have investigated melatonin as an anti-cancer agent; this molecule exhibits various functions in controlling the behavior of cancer cells, such as inhibiting cell growth, inducing apoptosis, and inhibiting invasion. The aim of this review is to comprehensively evaluate the role of melatonin as a complementary agent with 5-FU-based chemotherapy for cancers. Additionally, we identify the potential common signaling pathways by which melatonin and 5-FU interact to enhance the efficacy of the combined therapy.

**Graphic abstract:**

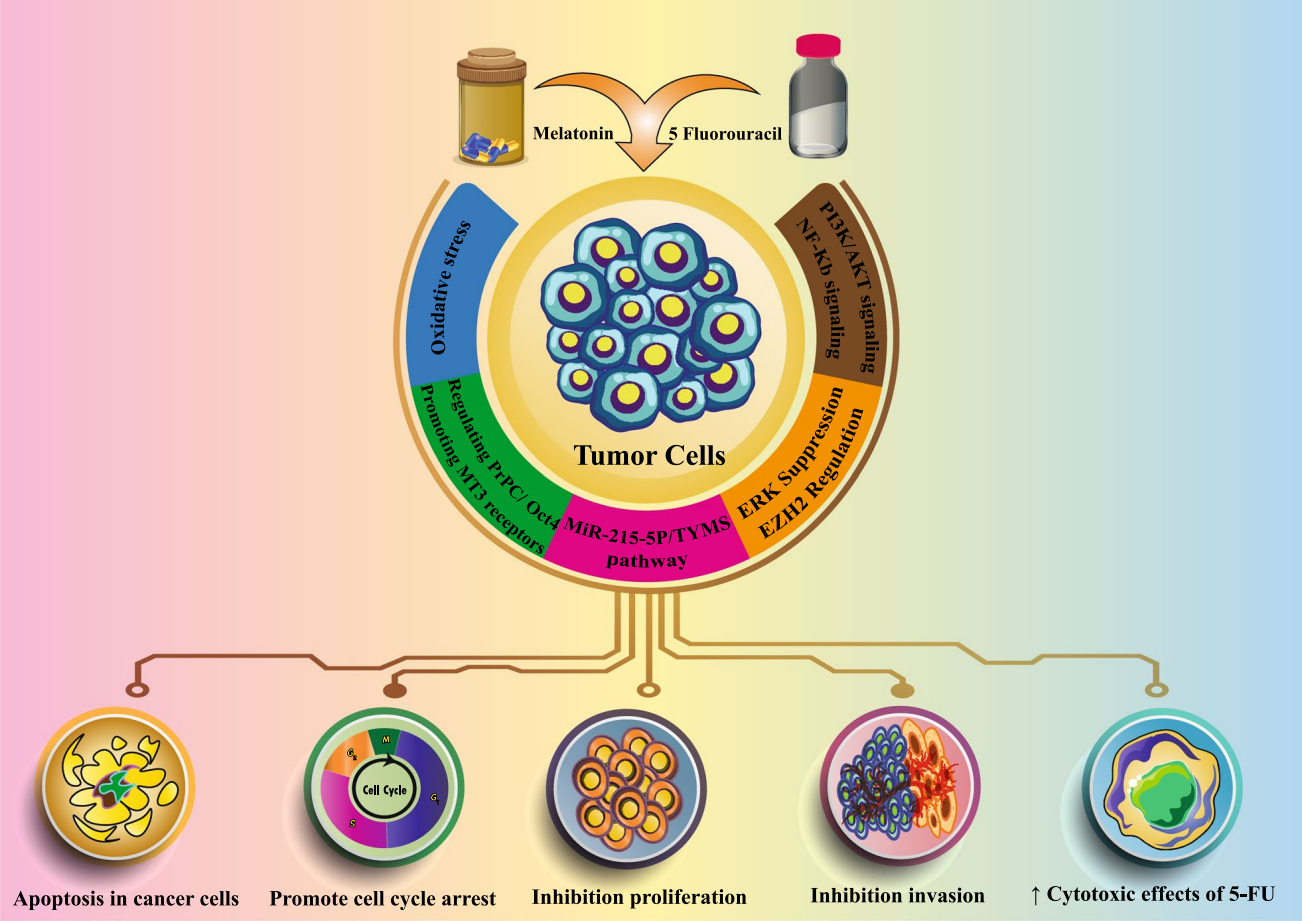

**Video abstract**

**Supplementary Information:**

The online version contains supplementary material available at 10.1186/s12964-023-01047-x.

## Introduction

Cancer is a general term used to describe a group of chronic diseases that result from abnormal proliferation of cells and can occur in all organs [1]. Pre-cancerous cells transform into malignant tumor cells in a progressive process. Cancer is the leading cause of death worldwide and has been a major burden for the World Health Organization for many decades. There were 23.6 million diagnosed cancer cases in 2019 and approximately 10 million deaths (one in six total deaths) due to cancer in 2020 alone. Cancer is a multifactorial disease in which genetics plays an important role and the incidence of cancer increases dramatically with age. There are more than 100 types of cancer; the most common types worldwide are: breast cancer, lung cancer, colon and rectal cancer, prostate cancer, skin cancer and stomach cancer [1, 2].

Since cancer has such a major impact on human health, it is important to identify effective and efficient treatments. Correct diagnosis of cancer is a prerequisite for optimal treatment, since each cancer type requires specific treatment. Surgery, radiotherapy, chemotherapy and hormone therapy are common means to treat cancer. In addition to the type of cancer, the patient's situation is an important factor in choosing the best treatment option. Chemotherapy has been one of the most common cancer therapies since the 1940s and significant progress in identifying more effective anti-cancer agents. Chemotherapy involves the administration of cytotoxic drugs to kill abnormal cells and destroy malignant tissue [3]. Usually, chemotherapeutic drugs are administered intravenously to maintain high levels of the drug and to improve safety. In some cases, the cytotoxic drug is injected directly into the affected tissue for better efficacy and fewer side effects.

Chemotherapeutic agents are categorized into several groups based on their mode of action. Alkylators and alkylator-related agents, as well as platinum agents, bind to intracellular macromolecules (such as DNA) and interfere with their function. Antimetabolites resemble essential molecules of the cell and interfere with the function of DNA and RNA, by mimicking them. Topoisomerase inhibitors suppress enzymes related to DNA transcription, i.e., topoisomerase I and II. Agents interacting on microtubules disrupt and destroy the protein skeleton of the cell. Fludarabine and cladribine resemble antimetabolites and induce programmed cell death (apoptosis) [4].

Multi-drug resistance (MDR) is common limitation in chemotherapy. Several mechanisms of drug resistance have been proposed. Many of these mechanisms involve an action at the cellular level to neutralize chemotherapeutic agents; increased metabolism and the excretion of drugs are other causes of drug resistance. Also, poorly vascularized tumors often exhibit drug resistance. One means to resolve or reduce drug resistance may be the use of combination chemotherapies; these treatments involve the combination of different types of drugs that either act on different metabolic pathways, or the combination of chemotherapies include pro-drug agents to increase the efficacy of primary chemotherapy. Combination chemotherapy was first introduced in 1960s; this was the MOPP regimen used to treat Hodgkin's lymphoma. While combination chemotherapies sometimes result in more efficient treatment strategies, they often lead to more side effects [5].

Melatonin is an endogenous molecule produced by many cells. The pineal gland, an outgrowth of the epithalamus, is the main source of circulating melatonin. The secretion of melatonin is mainly regulated by the light–dark cycle; thus, melatonin is one of the most important regulators of circadian rhythms of all organs. Besides, the production of melatonin by many extrapineal tissues and the presence of its receptors in various tissues have been identified. Further evidence demonstrated that the extrapineal melatonin is a multifunctional molecule and performs many other functions [6]. For example, it modulates the immune system and regulates T/B cells function and cytokine production. Many studies have documented the anti-inflammatory effects and potent antioxidant actions of this ubiquitously-acting molecule. Melatonin is also associated with bone homeostasis and has been used to treat osteoporosis. Over the last several decades, the anti-proliferative effects of melatonin against cancer cells have been extensively examined [7]. Melatonin is a cell-protective agent and is considered an anticancer agent due to its anti-proliferative, antioxidant, and immunomodulatory actions. In this review, we examine the current findings and the results of studies that show that melatonin may act as a worthy combination therapy when used with 5FU-based treatment. We also highlight the clinical roles of melatonin that may provide enhanced antitumor effects in combination with chemotherapy drugs.

## Melatonin

Melatonin (N-acetyl-5-methoxytryptamine), an indolamine neurohormone, is derived from serotonin, an intermediate compound, that synthetized from amino acid tryptophan, through intracellular enzymes such as tryptophan hydroxylase, aromatic amino acid decarboxylase, serotonin N-acetyltransferase, and hydroxyindole-O-methyltransferase [8]. Melatonin is considered as a highly preserved molecule produced by various spectrum of living organisms from bacteria to mammals [9, 10]. Melatonin is well known as a photosensitive hormone and important regulator of the circadian rhythm that produced by pineal gland [9]. However, accumulating studies revealed that many other tissues and organs, such as retina, gastrointestinal tract, lymphocytes of the skin, platelets, bone marrow, liver, heart, kidney, thymus, and cerebellum, are able to produce melatonin, called as extrapineal melatonin [6], which can play various essential roles in cellular processes via intracrine, autocrine and paracrine functions [11].

Melatonin synthesized in cells other than the pineal gland is not released in the blood circulation. It is release from the mitochondria into the cytosol and acts on receptors on the mitochondrial membrane [12]. The anti -oxidant activity of melatonin in the mitochondria of normal cells is mediated by two mechanisms: [1] a direct scavenger of free radicals and reactive oxygen species (ROS) and [2] an indirect function by increasing the expression and activity of endogenous antioxidant enzymes [13, 14]. The key role of melatonin in detoxification of free radicals during the cellular processes and metabolism and decreasing the oxidative stress is well describe in all organisms [15]. Additionally, melatonin plays an important role in promoting the immune system and postponing cell aging [16–18]. For example, it has been reported that melatonin that produced by thymus could regulate the production of some thymic peptides and prevents apoptosis of thymocytes [19, 20]. Also, it should be noted that melatonin can exert either a pro- or anti-inflammatory regulatory function in non-tumor cells under different conditions that is poorly understood. The stimulatory roles of melatonin in inflammation could be stimulated by induction of pro-inflammatory cytokines and other mediators. In contrast, melatonin can suppress inflammation by inhibiting NO release, inactivating toll-like receptor-4 and mTOR signaling, inhibiting pro-inflammatory cytokine release, enhancing release of the anti-inflammatory cytokines such as IL-4 and IL-10, and promoting the production of macrophage M2 that has anti-inflammatory action [21].

Recently, growing evidence identified that melatonin could act as an anti-cancer agent through suppressing cancer cell growth, proliferation, inducing apoptosis in cancer cells, and modulating the immune responses. Melatonin causes a deterioration in the T-cell populations in tumor microenvironment, with increased the CD8 + lymphocytes and decreased the T-reg numbers [22, 23]. Moreover, in cancer cells, melatonin shows pro-oxidant/cytotoxic effects, which depends on the pro-apoptotic signaling pathways such as NF-κB activation, leading to destroying the cancer cells [24–26].

## Antitumor activity of melatonin

Melatonin's anticancer activities have been confirmed by increasing evidence from experimental investigation. These anticancer function of melatonin, as well as the biochemical pathways involved, have been comprehensively demonstrated on tumor initiation, growth, and development [38, 39]. Melatonin is shown to suppress tumor growth and cancerous cells proliferation by prompting apoptosis in cancer cells, thus decreases the probability that healthy cells may develop neoplasms. It also promotes cellular turnover and the replacement of tumor cells with healthy cells via apoptosis [40, 41]. Melatonin is a potent antioxidant that prevents ROS-induced activation of Akt and extracellular-regulated protein kinase (ERK) pathways that are participated in the survival of tumor cells. Suppression of ROS-dependent Akt signalling pathway leads to reduction of cyclin D1 and Bcl-2 and elevation of Bax in cancerous cells [42]. Furthermore, melatonin affects the expression of MDM2, an E3 ubiquitin ligase that negatively regulates the p53 tumor suppressor, which results in the induction of apoptosis. According to the studies, melatonin through decreasing MDM2 expression promotes upregulation of caspase-3 and -9 activity and thus induces apoptosis [43, 44]. Additionally, melatonin causes apoptosis by simultaneously suppressing the p300/NF-B, COX-2/prostaglandin E 2 (PGE2), and PI3K/Akt signaling pathways. When these pathways are inhibited, Apaf-1 expression is increased, which causes the releasing of cytochrome c as well as the activation of caspase-3 and -9 [45]. Numerous investigations have shown that melatonin has anticancer effects in leukemia and solid tumors, with particular effectiveness in lymphoproliferative malignancies [46–48]. Bejarano et al. showed melatonin has proapoptotic and/or oncostatic action in HL-60 human myeloid cells by activating proapoptotic proteins such as Bax and Bid [47].

Several studies have shown that melatonin's antiproliferative effects are achieved via inhibiting or halting the cell cycle [49]. El Missiri et al. investigated the effect of melatonin on Ehrlich ascites carcinoma (EAC) cells. In this study, it was discovered that melatonin can cause the death of EAC tumor cells in addition to prolonging the survival of experimental animals [50]. Melatonin by influencing cell cycle-related proteins, such as decreasing the expression of cyclin D1, causes the inhibition of the proliferation and induces cell cycle arrest of HepG2 and Hep3B cell line of hepatocellular carcinoma (HCC) [51].

It has been demonstrated that melatonin modulates the angiogenesis process [52]. Under hypoxic situations in prostate cancer cells, melatonin suppresses the generation of HIF-1 by preventing the production of ROS and the sphingosine kinase 1 (SPHK1) pathway [53]. Melatonin also inhibits the growth of gastric cancer cells by regulating the TGF-1 signalling pathway, which is crucial in the regulation of tumor cell growth [54]. Melatonin inhibits the expression of endothelin-1 (ET-1) mRNA in colon cancer cells. ET-1 is a peptide that stimulates cell proliferation and angiogenesis in colon cancer. Melatonin inhibits the activity of the ET-1 promoter and thus decreasing ET-1 expression and production, which is regulated by NF-kb and FoxO1 [55]. Melatonin function as a natural antioxidant against oxidative stress has been demonstrated in various in vitro and in vivo research [56]. Melatonin detoxification of oxidants is caused by numerous processes. Melatonin not only neutralizes oxidants by scavenging free radicals, but it also promotes oxidative content reduction through a variety of mechanisms including activating antioxidant enzymes and inhibiting pro-oxidative enzymes. In addition, by stabilizing the mitochondrial inner membrane, the integrity of the mitochondria is preserved, and as a result, electron leakage and ROS production are reduced [57, 58].

Melatonin, an antioxidant, can elevate ROS production in specific types of cancerous cells because of the distinct differences between the metabolism of tumor cells and healthy cells (particularly glucose metabolism and cellular respiration) [59]. There are suggested two potential explanations for how melatonin enhance the level of ROS in cancer cells: First, melatonin directly acts as a stimulant ROS; this process happens when melatonin stimulates electron transport chain (ETC) complexes, causing them to generate free radicals and ROS by ETC complexes (I and III) [60, 61], and second, by reducing the number of antioxidant molecules that destroy ROS, melatonin disrupts the balance of oxidant and antioxidant compounds and cellular redox and thereby increases oxidative stress. According to research findings, melatonin decrease antioxidant enzymes and also rises lipid peroxidation in various types of cancer cells [62–64] whereas it reduces oxidative stress damage and enhances levels of activities of antioxidant enzymes in non-tumor cells [65].

Autophagy is modulated by melatonin in a variety of cell types and physiological situations. Melatonin affects oxidative stress and inflammation to modulate autophagy [66]. Melatonin increases the efficacy of cisplatin and radiotherapy in treating head and neck squamous cell carcinoma; this effects is caused by extreme mitochondrial induction that results in an excess of ROS and the consequent stimulation of autophagy and apoptosis [67]. Administering melatonin in mice model with colitis-associated colon carcinogenesis (CACC), by improving inflammation and oxidative stress, and on the other hand modulating autophagy and Nrf2 signaling pathways, reduced the process of autophagy and the progression of CACC [68].

## 5FU as a chemotherapeutic drug: from history to mechanisms of action

5-fluorouracil (5-FU) is a pyrimidine derivative used as an antimetabolite and is one of the most commonly used chemotherapeutic agents since the 1960s. Antimetabolites enter cells and mimic molecules critical for cell growth. They are naturally cytotoxic and disrupt the normal mechanisms of the cells by injuring the genetic material: DNA and RNA [69]. 5-FU is the major drug used in chemotherapy regimens for a variety of carcinomas such as gastrointestinal, breast, head and neck cancer, and skin cancer. Antibacterial (against *S. aureus* and *S*. *epidermidis*) and antiviral effects of 5-FU have also been observed in several studies [70].

5-FU is an analogue of uracil and differs structurally from uracil by having an additional fluorine atom attached to carbon 5 instead of a hydrogen atom. At room temperature, 5-FU is a colorless in a solution buffered with NaOH and it is administered intravenously [71]. Dihydropyrimidine dehydrogenase (DPD) is the main enzyme that degrades FU and is mainly present in the liver. 80% of 5-FU is immediately converted to inactive metabolites and 5–20% is excreted by the kidneys. Only 1–3% of administered 5-FU produces its clinical effects. Due to its short half-life (less than 20 min), 5-FU rapidly enters into cells after administration. Like uracil, 5-FU is transported into cells by the human nucleoside transporter (hNT). The human nucleoside transporter includes three concentrative constituents (hCNT 1,2,3) and four equilibrated transporters (hENT 1,2,3,4). 5-FU is transported only by hENT 1 and 2. In the cell, 5-FU is converted by phosphorylation into three major active metabolites: fluorouridine triphosphate (FUTP), fluorodeoxyuridine triphosphate (FdUTP), and fluorodeoxyuridine monophosphate (FdUMP). These active metabolites induce the anticancer effects of 5-FU via different pathways. The next section explains the proliferative inhibitory effects of 5-FU at the molecular level [72].

### 5-FU metabolites and DNA damage

Fluorodeoxyuridine monophosphate (FdUMP) impairs DNA repair and replication by inhibiting thymidylate synthase (TS) and thymidine formation. Thymidine is a crucial molecule for DNA repair and replication. Thymidylate synthase (TS) is an important enzyme for thymidine production. TS converts deoxyuridine monophosphate (dUMP) to deoxythymidine monophosphate (dTMP) using 5, 10-methylenetetrahydrofolate (CH2THF) as a methyl donor. Thus, it functions optimally as a complex with dUMP and CH2THF. FdUMP interferes with this complex by binding to TS in the nucleotide site (the site of dUMP) and impedes dTMP production. This leads to an imbalance of the deoxynucleotide pool with low dTTP and high dUTP concentrations, which eventually causes DNA repair cessation. Fluorodeoxyuridine triphosphate (FdUTP) (another active metabolite of 5-FU with anti-proliferative activity) is a substrate of DNA polymerase. FdUTP mis-incorporates with dUTP into DNA and damages its structure [69, 72, 73].

### 5-FU Effects on RNA

Initially, researchers believed that the main anti-cancer effects of 5-FU came from its destructive effects on DNA, but recent studies have shown otherwise. A study on the effects of 5-FU on U2snRNA in vivo found that metabolites of 5-FU are incorporated into U2 snRNA at pseudouridylation sites, inhibiting pre-mRNA splicing and pseudouridation of U2 snRNA. Several studies have demonstrated the inhibitory effect of 5-FU on the conversion of uridine to pseudouridine, resulting in defective and nonfunctional substance of pseudouridine-containing tRNA, mRNA, and snRNA. Fluorouridine triphosphate (FUTP) (active metabolite of 5-FU) can also interfere with RNA function. It mislocalizes to the rRNA precursor and inhibits proper transcription and maturation of rRNA. Thus, these studies demonstrate that the major proliferation inhibitory effects of 5-FU result from its incorporation into various RNA species [72, 74].

5-FU was approved as a drug by the FDA in 1962 and has since been widely used in various chemotherapy regimens, alone or in combination with other drugs. Several studies reported a marked effect of 5-FU especially in colorectal carcinoma, and the use of 5-FU in a combined chemotherapy regimen may have an efficacy rate of up to 40–60%. Despite the remarkable effect of 5-FU in cancer treatment over the past 60 years, drug resistance is a major limitation for the use of 5-FU, while the administration of 5-FU solely as chemotherapeutic regimen, showed a low response rate of 10–15% [73, 75].

Many studies have tried to identify mechanisms to overcome drug resistance of tumors. Chemoresistant cancer cells (CCCs) are a subclass of tumor cells that are not susceptible to chemotherapeutic agents. It has been suggested that CCCs develop from cancer stem cells and become refractory to 5-FU after altering some basic properties such as metabolism and proliferation [76]. To prevent and reduce 5-FU resistance, various modulations in chemotherapy have been proposed. Leucovorin (LV) or folinic acid is an analog of folic acid and is used to reduce the toxicity of metotroxate. LV increases the intracellular concentration of CH2THF by converting to it and enhances the ternary complex of CH2THF, thymidylate synthase, and FdUMP. As a result, the toxicity and proliferation inhibitory effects of 5-FU increase. Dihydropyrimidine dehydrogenase (DPD) is the main enzyme that degrades pyrimidines, uracil and thymine, and it is also responsible for the degradation of almost 80% of 5-FU immediately after its administration. The combination of uracil and the pro-drug 5-FU (ftorafur) as UFT (uracil/ftorafur; in a 4/1 ratio) is one of the strategies used to reduce the effects of DPD on 5-FU. Capecitabine is a chemotherapy drug used to treat breast cancer, gastric cancer, and colorectal cancer. It is administered orally and converts to 5'-deoxy-5-fluorouridine (5'DFUR) in gastrointestinal wall cells. 5'DFUR eventually converts to 5-FU, which actively kills the tumor cells [69, 72, 73].

## Melatonin and 5-FU combination chemotherapy in cancer

The efficacy and clinical application of 5-FU is limited by the development of drug resistance, which is common in cancer chemotherapy [77, 78]. The combination of 5-FU, as the main chemotherapeutics agent, with naturally occurring bioactive compounds, has recently gained attention as a means to reduce the effective dose, and thereby adverse side effects [79]. Regarding melatonin, a pleiotropic molecule with various biological activities, has increasingly emerged as an effective adjuvant agent to conventional anticancer therapy for a variety of cancer types [80, 81]. Reduction in drug resistance to antineoplastic agents and chemotherapy-induced toxicity are other benefits of melatonin application in clinical oncology as has been a greater therapeutic response [82–85]. In addition to the diverse range of proven biological functions of melatonin in oxidative stress (anti-oxidative effects) [65], and immunomodulation (anti-inflammatory effects) [55, 86], the involvement of melatonin in anticancer and oncostatic processes including apoptosis induction, as well as inhibition of cell proliferation, tumor growth, and metastases has been repeatedly confirmed [87, 88].

### Induction apoptosis via pro-oxidants activity

As a follow-up investigation, an experimental study conducted by Uguz et al. investigated the augmenting effects of melatonin on the efficacy of chemotherapeutic drugs, which included 5-FU. Their results demonstrated that melatonin and 5-FU co-treatment in AR42J cells of rat pancreatic cancer model, resulted in increased apoptosis via induction the mitochondrial membrane depolarization and ROS production in the cancer cells, leading to an enhancement of the chemotherapy-induced cytotoxicity effects, compared to treatment with 5-FU alone [85]. Previously, the findings of several studies suggested that melatonin may enhance the efficacy of other current chemotherapeutic drugs in terms of tumor regression via potentiating their chemotherapy-mediated cytotoxicity [90, 91] and promoting apoptosis [92, 93], which led to an increased survival rate of cancer-bearing humans [94]. An elevated intracellular ROS production and the subsequent loss of mitochondrial membrane potential; this resulted in the activation of mitochondrial apoptogenic factors, such as cytochrome c release, with the stimulation of the caspase cascades which are fundamental mechanisms of the intrinsic apoptosis pathway [95, 96].

Mihanfar et al. [97] reported that melatonin in combination with 5-FU significantly decrease the IC50 value of 5-FU from 50 to 100 μM as well as improved the cytotoxic efficacy of 5-FU inSW-480 CRC cell line of human CRC. Regarding the underlying mechanism, melatonin negatively regulated the resistance of cancer cells to apoptosis through targeting of oxidative stress, X-linked IAP (XIAP) and survivin in CRC cells, hence decreasing the cell proliferation. Indeed, the intracellular levels of ROS increased, and in contrast, the antioxidant enzymatic activities, and the expression levels of XIAP and survivin were downregulated after combination therapy of melatonin and 5-FU [97]. Selective apoptosis induction in CRC cells is an important anti-neoplastic function of 5-FU [98, 99]. Moreover, dysregulation of apoptotic pathways was proven to be potently related to failure of cancer treatment and to drug resistance [100–102]. XIAP and survivin are recognized as major members of the inhibitor of apoptosis proteins (IAPs) family, which are involved in anti-apoptotic processes through suppression of caspase activity [103–105]. Invariably, the essential function of XIAP and survivin in determining the resistance of cancer cells to apoptosis and subsequently to anti-neoplastic agents were demonstrated in various human malignancies; thus, it appears that downregulation of IAPs may contribute to successful treatment via improving of the sensitivity of cancer cells to chemotherapy and/or radiotherapy mediated by regulation of apoptosis [103, 106–109]. In this regard, a recent report noted that double knockdown of survivin and XIAP enhanced the sensitivity of human CRC to radiation therapy and also mediated a reduction in the cancer cells migration [110]. Additionally, melatonin in combination with 5-FU elevated Bax expression as well as Bax/Bcl-2 ratio in CRC cells [97] and the phosphorylation of Bcl-2 and Bax; as members of the Bcl-2 family they participate in promoting the p53-mediated apoptosis in cancer [111, 112]. The effect of the combination of melatonin and 5-FU for improvement of 5-FU potency for CRC treatment may be in part mediated by ROS overproduction and reduction of antioxidant levels in cancer cells [97]. Certainly, recent evidence indicates that the anti-cancer effects and adjuvant actions of melatonin as a co-treatment with other chemotherapeutic drugs involves ROS overproduction and a reduction of antioxidant enzymes, leading to increased apoptosis [64, 113]. It is known that ROS play dual roles in cancer; while at low levels, ROS can exert oncogenic activity [114], at high levels they provide an intracellular oxidizing environment leaing to induction of apoptosis [115]. One report documented that melatonin intensifies the anti-cancer effects of 5-FU and cisplatin in human colorectal adenocarcinoma HT–29 cells, by moderating the chemo-sensitivity and proapoptotic processes. This study revealed that in the presence of melatonin further increased caspase-3 and caspase-9 activation following FU-5 application. Also, melatonin promoted 5-FU-evoked ROS production, and thereby enhanced mitochondrial apoptosis of cancer HT–29 cells [113].

### Induction apoptosis via PI3K/Akt and Erk signaling pathway

In similar studies, combined treatment with melatonin and puromycin, a chemotherapeutic agent, caused a dysregulation of the cell cycle and promoted the pro-apoptotic activities of puromycin by enhancing the downregulation of the anti-apoptotic proteins, such as Bcl-2 and Bcl-xL, activation of caspase-3, poly-(ADP-ribose) polymerase (PARP), and 5′-adenosine monophosphate-activated kinase (AMPK) [116]. Moreover, several studies indicate that melatonin in combination with conventional anti-cancer drugs significantly increases apoptosis via several mechanisms including activation of caspase-mediated and inactivation of Erk/p90RSK/HSP27 cascades in cancer, without any measurable changes in normal non-cancer cells [93, 117]. Indeed, it has been repeatedly demonstrated that melatonin has a cytoprotective effects in normal cells against chemotherapy-induced cytotoxicity, apoptosis, and genomic damage [90, 118, 119], actions that may be mediated by its antioxidant properties [57].

Considering the findings mentioned in the previous paragraph, it was not surprising when similar results were reported in which melatonin notably enhanced 5-FU-mediated inhibition of cell proliferation, migration and invasion of SW620 and LOVO cells in mouse model of human CRC. Further mechanistic investigations revealed that melatonin synergized with 5-FU efficacy by simultaneous inhibiting multiple signaling pathways including activation of caspase/PARP-dependent apoptosis, induction the cell cycle arrest, suppression of the PI3K/Akt and NF-κB/iNOS signaling pathways, inhibition of MMP9 expression, and on the contrary promotion of E-cadherin expression [120]. NF‑κB, a transcription factor, exerts an important role in regulation of anti-apoptotic proteins and growth factors, and thereby promotes tumorigenesis by elevating the expression of nitric oxide synthase (iNOS) [121, 122]. In this sense, accumulating evidence indicates that inducible iNOS is markedly upregulated in certain inflammatory and cancerous tissues [123–125], and also capable to promote tumor development and progression [126, 127]. Moreover, the anti-tumor activity of melatonin is likely mediated in part by downregulation of iNOS expression [128, 129]. The PI3K/Akt signal transduction cascade possess anti-apoptotic activity; hence, inhibition of this pathway participates in apoptosis induction [130]. The PI3K/Akt signaling is also a target of melatonin [131, 132]. The results of a recent study indicated that survival of melanoma cells after co-treatment with melatonin was suppressed through regulation of the PI3K/Akt/mTOR pathway [133]. Moreover, increasing the expression of E-cadherin and reducing the expression of MMP9 contribute to the development of chemotherapeutic drug resistance, metastasis and cancer invasion [134, 135].

As with other investigators, Lu et al. [136]. assessed the impact of co-treatment with melatonin on the sensitivity to 5-FU in esophageal squamous cell carcinoma (ESCC) in a mouse model. They concluded that melatonin improved the suppression of cell proliferation, migration, invasion, and promoted apoptosis in a mitochondria-dependent manner in ESCC cells in vitro, as well as inhibition of tumor growth in vivo [136]. A probable underlying mechanism of melatonin relates to its association with the regulation of extracellular regulated protein kinase (Erk) and protein kinase B (PKB or Akt). Indeed, co-treatment with melatonin reverses the impact of 5-FU on the of activation MEK/Erk and GSK3β/Akt signaling pathway, which effectively leads to increased sensitivity to 5-FU in ESCC cells, as well as the cytotoxicity of 5-FU in ESCC [136]. Akt, activated by PI3K, enhances tumorigenesis via regulation of phosphorylation of several downstream target genes including GSK3β and mTOR [137, 138]. Phosphorylation-mediated activated Akt is over-expressed in various type tumor, which is associated with poor prognosis [138–140]. The genetic abnormalities in the PI3K/Akt signaling pathway is common in human cancer [137]. Also, genetic variations in PI3K/PTEN/Akt/mTOR axis were found to be predict the elevated recurrence risk of esophageal cancer after chemoradiotherapy [141]. Conversely, the overexpression of an activated Erk pathway and its role in cell proliferation and progression of head and neck squamous carcinoma, such as in ESCC cells, was detected [142, 143].

It has been reported that Erk and Akt can cooperatively modulate downstream gene expression to cell proliferation and cell cycle progression [144]. Regarding the relationship between PI3K/Akt and the Erk pathway, applying PI3K inhibitors cause MEK/Erk pathway suppression in breast cancer cells. Also, inhibition of both Akt and Erk pathways are necessary for optimal antitumoral effects [145]. Finally, it has been documented that suppression of Erk and Akt pathways are, in part, responsible for tumor-suppressive functions of melatonin in breast cancer [45].

### Stimulation of MT3 receptor

Pariente et al. [146] documented that melatonin potently enhanced the cytotoxic and apoptotic effect of chemotherapeutic agents including cisplatin and 5-FU in human CRC colon HT-29 cells and cervical cancer HeLa cells; this was particularly obvious in the 5-FU-challenged cells. Indeed, melatonin can to be effective in terms of promoting the tumor cell sensitivity of 5-FU through the signal transduction elicited by MT3 receptor stimulation [146]. In addition, concomitant treatments with melatonin and 5-FU cause further increased caspase-3 activation, thereby evoking the 5-FU-mediated apoptotic cell death, which also is mediated by MT3 receptor trigger [146]. MT3 is a melatonin receptor that is present in the retina, liver, heart, intestine, kidney, muscle, and fat and acts as a fluid pressure regulator and detoxification enzyme. It may have neuroprotective and oncostatic effects. [28, 29].

### Regulate cancer stem cells

Investigations conducted by Lee et al. [147] revealed that the combination of 5-FU and melatonin inhibits proliferation, promotes apoptosis and autophagy of colon cancer stem cells (CSCs), thereby suppressing the CRC progression and tumor-mediated angiogenesis; this involves targeting the PrPC-Oct4-HSPA1L axis. 5-FU and melatonin suppressed the expression of human CSC markers, particularly Oct4, by downregulating cellular prion protein (PrPC) expression, Also, PrPC and Oct4 expressions were found to be associated with human CRC metastasis [147]. The findings of this study also suggested that PrPC prevents Oct4 degradation by enhancing the binding of heat shock protein family A member 1-like (HSPA1L) to octamer-binding transcription factor 4 (Oct4) [147]. CSCs, which are capable of self-renewing, account for tumor development and maintenance, as well as treatment failure [148]. Oct4 acts is an essential transcriptional factor for self-renewal, pluripotency, and survival of CSCs, and reprogramming [149], through a potential Oct4-AKT-ABCG2 regulatory circuit [150]. Also, Oct4 gene knockdown induces apoptosis of CSCs, leading to suppressed tumor growth [151], and weakened tumorigenicity of drug-resistant cancer cells [152]. For example, pancreatic cancer cells resistant to 5-FU overexpressed the Oct4 gene [153]. The metastatic and angiogenic roles of Oct4 have also been described [154]. Growing evidence indicates that PrPC takes part in behaviors of cancer cells such as, invasion, metastasis, cell migration, proliferation and apoptosis and finally tumor survival and progression [155–157]. In addition, PrPC participates in cancer cell self-renewal [158]. In addition, PrPC found to be associated with chemoresistance [159]. The expression of heat shock proteins (HSPs), which are correlated to tumor cell proliferation and the inhibition of apoptosis [160], recently have been found to be associated with Oct4 and PrPC expression in tumor cells [161–163]. Melatonin administration promotes senescence, autophagy, and apoptosis in human CRC cells [164]. An earlier study indicated that melatonin advances apoptosis in oxaliplatin-resistant CRC cells through inhibition of PrPC [165].

### Regulate immune response

A clinical trial, the results of which were published in 1995, reported that low-dose interleukin-2 (IL-2) and melatonin prolonged the one-year survival and improved the quality of life in patients with metastatic colorectal cancer (CRC), who progressed despite initial response to 5-FU [89]. In 2018, Akyuz et al. [166]. observed that treatment with melatonin improved healing following colonic anastomosis and attenuated the adverse effects of pre-operative chemotherapy with 5-FU. Histopathological examination exhibited remarkable reduction in inflammation and necrosis formation in the rat model group treated with melatonin [166]. This study also showed that serum TNF-α and IL-1β levels were significantly reduced, leading to decreased the activation and infiltration of neutrophils, which have a main role in inflammatory tissue damage, after melatonin treatment [166]. It is known that TNF-α and IL-1β play a major regulatory roles in the inflammatory response and their cytotoxic effects [167]. Under inflammatory and trauma conditions, neutrophils are stimulated and release ROS and cytokines, such as TNF-α and IL-1β [168].

### Epigenic regulation

Importantly, an evaluation of the effect of melatonin on molecular mechanisms in 5-FU resistant human CRC cells provided significant results. Significant additive effects of conjugation of FU-5 therapy with melatonin led to inhibition of the cell growth, promotion of apoptosis, enhancement of 5-FU-mediated cytotoxicity and sensitivity of 5-FU resistant CRC cells. As an explanation of the underlying mechanism, these effects were manifested through depressing thymidylate synthase (TYMS) transcription and expression, which were mediated by upregulation of miR-215-5p. Hence, TYMS serves as the key direct downstream target for miR-215-5p [169]. TYMS, a substantial target for chemotherapeutic agents and a known folate-dependent catalytic enzyme, exhibits substantial effects on the production of intracellular thymidine, a fundamental precursor for DNA biosynthesis [170, 171]. Accumulating evidence has demonstrated that TYMS expression inversely associates with 5-FU sensitivity and efficacy in cancer cells. Therefore, TYMS is suggested as an important determinant for therapeutic responsiveness to 5-FU and mechanistic driver of 5-FU resistance [172–174]. Moreover, high expression of TYMS in tumor tissues indicates poor responsiveness to 5-FU, and thereby worse overall prognosis [175–177]. Previously, it was reported that TYMS mRNA includes several miRNAs, including miR-433 and miR-203 that negatively regulate TYMS expression; this in turn, promotes 5-FU chemosensitivity of cancer cells [178, 179]. Melatonin was also found to exert antitumor and anti-oxidant effects by regulating the expression of several miRNAs [180–182]. Additionally, the tumor inhibitory function of miR-215 was found to target NOB1 in ovarian cancer [183] and by targeting AKT serine/threonine kinase 1in breast cancer [184]. Also, miR-215 is involved in the cellular 5-FU response via regulation of the TYMS expression in tumor cells [185, 186].

### Circadian changes

Baldueva et al. [187] demonstrated that oral melatonin supplementation improved the efficacy and the antitumor response to chemotherapy regimens including cyclophosphamide, adriamycin, 5-FU, and docetaxel, in HER2/neu transgenic mice model with spontaneous mammary adenocarcinomas; these therapies had been shown to be ineffective perhaps related to the inapropriate time of administration. Also, melatonin supplementation reduced toxicity and provided further improvement of cancer stabilization rates [187]. Involvement of periodic cellular gene expression by the circadian system is controlled by chronobiotic agents such as melatonin; these coordinate cellular physiology with diurnal alterations in the environment [188]. The circadian changes in cell biology influence the cellular processes (including metabolism, signaling networks) as well as have an impact on the molecular processes such as DNA repair and cell cycle in response to different treatments [189]. Since, circadian rhythms are disturbed in patients suffering from cancer, a chronobiotic such as melatonin can influence tumor responsiveness to chemotherapy drugs as well as modulating the chronopharmacokinetics and chronopharmacodynamics of anticancer agents [190–193]. Also, recent findings revealed that tumor glucose metabolism may be switched from cytosolic glycolysis to mitochondrial oxidative phosphorylation by the nighttime rise in circulating melatonin [194].

### Inhibition of oncogenic factors

Similar results were obtained by Zhang et al. [195] in which the synergistic effects of melatonin and 5‑FU in combination therapy for human esophageal cancer (EC) were apparent. In this case, melatonin improved the sensitivity to 5-FU and diminished the IC50 of 5‑FU in EC cells [195]. The 5‑FU‑mediated inhibition of the cell proliferation, migration, and invasion, as well as activation of cell apoptosis in EC‑9706 and EC‑109 cells was strongly enhanced when melatonin was added with 5-FU; hence, melatonin sensitizes EC cells to 5-FU [195]. The data collected from this study also showed that melatonin and 5-FU co-treatment exerted an anti-proliferative and pro-apoptotic function through downregulating of histone‑lysine N‑methyltransferase EZH2 (EZH2) expression. In addition, it is known that EZH2 participates in EC via activation of the JAK2/STAT3 signaling pathway [195]. The expression of EZH2, an enzymatic subunit of polycomb repressive complex 2 (PRC2), is upregulated in various types of cancer cells, and also is under the influence of multiple oncogenic factors. Therefore, EZH2 plays a critical oncogenic role in numerous related processes including cancer initiation, differentiation, development, progression, and metastasis [196–198]. Accumulating evidence has revealed the key contributing role of EZH2 in increasing the resistance of numerous cancer cells to chemotherapeutic drugs such as 5-FU [199–201]. The results of earlier studies also reported that melatonin may act as a tumor inhibitor molecule in glioblastoma stem-like cells by recruiting the EZH2-related axis, which could involve multiple pathways including EZH2‑NOTCH1 [202] and AKT‑EZH2‑STAT3 signaling [203].

## Conclusions

Research has shown that combination therapies may not only reduce drug resistance, but also can simultaneously provide improved anti-cancer effects such as by reducing cell proliferation, limiting tumor growth, promoting cell cycle arrest, and increasing apoptosis in cancer cells. It is noted, however, that these effects are not always apparent and this approach may only increase cancer cell toxicity, without having a substantial effect on controlling the behavior of cancer cells.

**Fig. 1:**
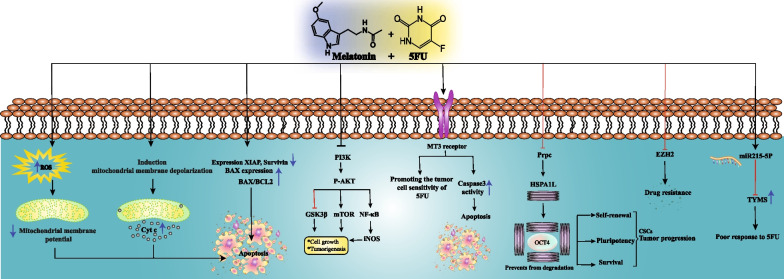
A schematic view of the combination therapy of melatonin and 5-FU in regulating the signaling pathways of cancer cells. The combination therapy of melatonin and 5-FU can lead to effects such as induction of apoptosis, increasing the sensitivity of cells to 5-FU and inhibition of cell growth through different signaling pathways. Red blocked-end arrow illustrate inhibition and black arrow show stimulation and/or activation. Blue down arrow: decrease, blue up arrow: increase. Abbreviations: ROS, Reactive oxygen species; XIAP, X-linked inhibitor of apoptosis protein; GSK3β, Glycogen synthase kinase 3β; NF-κB, Nuclear factor κB; iNOS, Inducible nitric oxide synthase; Oct4, Octamer-binding transcription factor 4 TYMS, Thymidylate synthetase

5-FU is a representative of the category of antitumor drugs and widely used chemotherapy in the management and control of cancer. In recent years, the potential of melatonin in the treatment and management of some disorders has increased because of its multiple actions as an antioxidant and as an anti-inflammatory molecule. Also, the antitumor effects of melatonin and its effects in increasing the efficiency of other therapeutic procedures such as chemotherapy, have been reported. Herein, we describe the extensive role of melatonin in combination with 5-FU in cancer treatment. The studies reviewed in this report show that melatonin may be an important cornerstone treatment for a variety of cancers, although in each case the specific mechanism have not been elucidated. Since conventional chemotherapies often have severe side effects and negatively influences the quality and outcome of life. Melatonin has also proven its benefits as an adjuvant to reduce the side effects of these toxic drugs; this makes melatonin a highly suitable ancillary treatment option. The combination treatment of 5FU with melatonin in both in vitro and in vivo cancer models increases the hope for a brighter future for the common use of combined chemotherapies. Overall, melatonin in combination with 5FU as a chemotherapy drug may improve its clinical application in cancer treatment and play a significant role as an adjunct for a variety of different tumors (Table 1 and Fig. 1). It is noted that the current evidence was mainly obtained in preclinical models of cancer, and for this reason, more studies are needed to better understand the therapeutic potential and the underlying mechanisms of melatonin combined chemotherapy. It is also necessary to further investigate the effects of melatonin in different cell signaling pathways to better define the mechanisms of these combined therapies.

**Table 1:** Melatonin enhancement the antitumor activity of 5-FU chemotherapy

Cancer Type	Model	Sample type	Dose of Melatonin	Signaling pathway	Findings	References
Pancreatic cancer	In vitro	AR42J cells	1 mM	–	Promotion the depolarization of mitochondrial membrane and ROS generation in cancer cells ↑ Apoptosis in cancer cells	[85]
Colon cancer	In vitro In vivo	SW620 and LOVO cells Female athymic nude mice	1 mM 25 mg/kg	PI3K/Akt signaling NF-κB/iNOS signaling	Suppress of proliferation, migration, invasion and colony formation in colon cancer cells Induce the activation of apoptosis via caspase/PARP- dependent pathway Promote cell cycle arrest	[120]
Oesophageal squamous cell carcinoma	In vitro In vivo	KYSE520, KYSE410, KYSE150, KYSE30 cells Female BABL/c nude mice	Range of 0–5 mM 25 mg/kg	Suppression of Erk and Akt pathway	Inhibition proliferation, migration, invasion and promotion apoptosis (mitochondria-dependent) of ESCC cells in vitro Inhibition tumor growth in mice model ↑ Effectively cytotoxicity of 5-Fu to ESCC in vitro and in vivo Inhibition the phosphorylation of Erk and Akt	[136]
Colorectal cancer Cervical cancer	In vitro	HT-29 cell line HeLa cell line	1 mM	Promotion of MT3 melatonin receptors	↑ chemotherapeutic-induced apoptosis and cytotoxicity in tumor cell lines	[146]
Colon cancer	Human In vitro In vivo	Colorectal cancer specimens SNU-C5/WT, SNU-C5/5FUR, SNU-C5/Oxal, S707 cells Xenograft models mice	–	Regulation of PrPC and Oct4 axis	Inhibition the colorectal cancer cell proliferation and development via promoting autophagy and apoptosis	[147]
Colorectal cancer	In vitro	HT29 cell line	1 mM	N/D	↑ Cytotoxic effects of 5-FU ↑ Sensitivity of cell line to treatment with 5-FU ↑ Ratio of cells that overproduce intracellular ROS and significantly increased the population of apoptotic cells	[113]
Anastomotic leakage	In vivo	Male Wistar rats	0.4 mg/kg	N/D	** ↓ Inflammation and necrosis formation ↓ TNF-α and IL-1β plasma levels	[166]
Colorectal cancer	Human In vitro	Cancer tissues HCT116, SW480, COLO320, DLD-1, HT29, RKO, CaCO2 and SW620 cells	Different dosage (1–4 mM)	Upregulation of miR-215-5p / simultaneously downregulation of TYMS	Inhibition the cell growth Induction apoptosis ↑ Cytotoxic effects of 5-FU in 5-FU resistant cells	[169]
Esophageal cancer	In vitro	EC-9706 and EC-109 and HET-1A cells	0–2 mM	Regulating EZH2 expression	Inhibition of cell proliferation Promotion of apoptosis Inhibition of invasion and migration in EC-9706 and EC-109 cells	[195]
Colorectal cancer	In vitro	SW-480 cell lines	75, 150, 200 µM	Increasing OS and targeting XIAP and surviving	↓ Cell proliferation ↑ Intracellular levels of ROS and Inhibition the activity of antioxidant enzymatic Reversing effect on the resistance to apoptosis and augmented the cytotoxicity of 5-FU	[97]

^*^ **↑** indicates the elevation

^**^ ↓ indicates the reduction

*ROS* reactive oxygen species, *ESCC* Esophageal squamous cell carcinoma, *PrPc* Cellular prion protein, *Oct-4* octamer-binding transcription factor 4, *TYMS* thymidylate synthase, *OS* oxidative stress

## Data Availability

Not applicable.
